# Blood pressure trend in hospitalized adult dengue patients

**DOI:** 10.1371/journal.pone.0235166

**Published:** 2020-07-01

**Authors:** Wesley Yeung, David Chien Boon Lye, Tun-Linn Thein, Yirong Chen, Yee-Sin Leo

**Affiliations:** 1 Yong Loo Lin School of Medicine, National University of Singapore, Singapore, Singapore; 2 Department of Infectious Diseases, Tan Tock Seng Hospital, Singapore, Singapore; 3 National Centre for Infectious Diseases, Singapore, Singapore; 4 Saw Swee Hock School of Public Health, National University of Singapore, Singapore, Singapore; 5 Lee Kong Chian School of Medicine, Nanyang Technological University, Singapore, Singapore; International Medical University, MALAYSIA

## Abstract

**Background:**

Monitoring of blood pressure is an important part of management of dengue illness. Large scale studies of temporal trend of blood pressure in adult dengue are lacking. In this study, we examined the differences in time trend of systolic (SBP) and diastolic blood pressure (DBP) in patients with and without severe dengue (SD), dengue hemorrhagic fever (DHF) and pre-existing hypertension, and elderly versus non-elderly patients.

**Methods:**

We studied a retrospective cohort from 2005 to 2008 of 6,070 hospitalized adult dengue patients confirmed by polymerase chain reaction or clinical criteria plus positive dengue serology. Dengue severity was defined according to World Health Organization 1997 and 2009 guidelines. We used Bayesian hierarchical Markov models to compare the daily mean SBP and DBP between different subgroups. Analysis was conducted by day of defervescence (denoted as day 0), and day of illness onset (denoted as day 1) respectively.

**Results:**

SBP decreased to a nadir during the critical phase before defervescence and was significantly lower for patients with SD or DHF, compared with patients without SD or DHF. DBP increased marginally more for patients with SD or DHF in the critical phase before defervescence. By day of defervescence, comparison of patients with and without SD showed significant difference in SBP from day -6 to day +6, except days +1, +3 and +5, and similarly in DBP except days 0, and +4 to +6. Comparison of patients with and without DHF showed significant difference in SBP from day -6 to day -1, but for DBP, significant difference was noted from day -6 to day +6, except day -2 to day 0. By day of illness, SBP differed significantly between patients with and without SD from illness days 1 to 10, and DBP from illness days 7 to 12. Between patients with and without DHF, SBP differed significantly on illness days 1, 2, 4 to 7, while DBP from days 7 to 12. On analysis by days of defervescence or by days of illness, elderly patients and those with hypertension showed consistently higher SBP and DBP throughout their hospitalization, as compared with their younger and non-hypertensive counterparts.

**Conclusion:**

In SD or DHF, SBP decreased to a nadir around the day of defervescence, and recovered to a level exceeding that in febrile phase by days 2 or 3 post-defervescence. Elderly patients and patients with pre-existing hypertension maintained higher SBP and DBP throughout the duration of dengue infection.

## Introduction

Dengue is a mosquito-borne viral infection with an estimated 390 million infections annually, of which 96 million are clinically apparent [1]. Despite aggressive vector control, Singapore continues to experience cyclical large dengue outbreaks affecting mostly adults. In the first six months of 2019, there were 7,353 reported cases of dengue in Singapore with 5 fatalities [2]. Dengue can range from an asymptomatic infection to a severe life-threatening systemic illness with circulatory failure and death. Severe forms of dengue, including severe dengue (SD) [3], dengue hemorrhagic fever (DHF) with or without dengue shock syndrome (DSS) [4], are uncommon but associated with a mortality rate of up to 20% if untreated [5]. These clinical entities are associated with increased capillary permeability and plasma leakage which manifest during a ‘critical phase’ around the time of defervescence, typically between days 4 and 7 of disease, and lasts for approximately 48 hours [6]. Plasma leakage leads to depletion of the intravascular compartment and hypotension. A pediatric study in Vietnam found that 7% of patients had unrecordable blood pressures at presentation while 26% of patients were hypotensive for age [7]. In adults, hypotension is highly associated with dengue-related deaths and severe disease [8–10].

Atypical clinical presentations and higher rates of SD among elderly patients were reported at our center [11] and elsewhere [12]. Pre-existing hypertension was found to contribute to a higher risk of severe illness in diabetic patients [13]. These risk factors represent certain high risk groups in the population. A high prevalence of these chronic conditions [14, 15] and an aging population in Singapore [16] and other countries [17, 18] where dengue is prevalent present additional challenges.

Although close monitoring of clinical and laboratory parameters coupled with timely fluid replacement is associated with lower mortality [5], data from large studies of vital signs including blood pressure are lacking. In this large cohort study of all adult dengue patients admitted to the Department of Infectious Diseases, Tan Tock Seng Hospital, Singapore from 2005 to 2008, we aimed to describe the temporal trend of systolic (SBP) and diastolic blood pressure (DBP) over the course of hospitalization. We studied four subgroups: (i) older adults aged above 60; (ii) patients with pre-existing hypertension; and patients who developed (iii) SD and (iv) DHF.

## Methods

### Ethics statement

This study was approved by the Domain Specific Review Board, National Healthcare Group, Singapore. Informed consent from patients was waived. All data were anonymised.

### Participants

This was a retrospective cohort study on all adult (≥18 years) dengue patients admitted to the Department of Infectious Diseases at Tan Tock Seng Hospital, Singapore, where all patients were managed with a standardized clinical care path from 1 January 2005 to 31 December 2008. Dengue infection was confirmed by either a positive reverse transcriptase polymerase chain reaction (RT-PCR) [19] or diagnosed as probable dengue by applying diagnostic criteria from World Health Organization (WHO) 1997 or 2009 guidelines coupled with a positive Panbio Rapid Dengue Duo Rapid Strip Test (Panbio Diagnostic, Queensland, Australia) [20, 21]. Blood pressure was routinely measured four times a day by the nurses using digital automatic blood pressure monitors. When necessary, the doctors confirmed the blood pressure by using mercury column devices. The records were made manually on charts. Clinically trained research assistants reviewed the demographic, clinical features, investigations and treatment data from medical records and entered into the Microsoft Access database.

### Clinical outcome

In this study, dengue hemorrhagic fever (DHF) and severe dengue (SD) were defined according to WHO 1997 [4] and 2009 guidelines https://paperpile.com/c/HsoZio/c4lC[3]. DHF was diagnosed when all four criteria of fever, thrombocytopenia ≤ 100 × 10 9 /L, hemorrhagic manifestations, and plasma leakage (hypoproteinemia, ≥ 20% change in hematocrit, or pleural effusion or ascites) were present [4]. We analysed all DHF patients with or without DSS. SD was fulfilled if there was evidence of plasma leakage associated with shock or respiratory distress, severe bleeding or severe organ involvement [3]. Elderly patients were defined as patients who were aged 60 years or older. Previous history of hypertension was defined as a self-reported clinical history of hypertension. Defervescence was defined as recorded body temperature ≤ 37.5°C for more than 24 hours. Febrile, critical, and recovery phases were represented by illness days 1–3, 4–7, 8–12. When the patients had multiple blood pressure measurements in a day, we took the minimum SBP reading and maximum DBP reading.

### Statistical analyses

Categorical variables were described by counts and percentages. Continuous variables were described by means and standard deviations (STD). Data analyses were performed under a Bayesian framework. Tests of proportions were used to compare categorical variables between groups using non-informative beta (1,1) priors. Bayesian estimation was used to estimate and populate parameters of each subgroup and compare differences in means of continuous variables using weakly informative normal priors.

Time trends of SBP and DBP were analyzed separately by illness day and defervescence day and presented in separate plots. Day of onset of fever was denoted as illness day 0. The day of defervescence was denoted as day 0, day before defervescence as day -1, and day after defervescence as day 1 respectively. The daily means of the SBP and DBP in each subgroup were modeled using hierarchical Markov models with homoskedastic stochastic innovations, homoskedastic errors, with random effects assumed to act multiplicatively on the grand mean. The data model wa *y*_*i*_(*t*_*j*_) ~ *N*(*β*_*i*_*b*_*j*_,σ^2^) where *y*_*i*_(*t*_*j*_) is the measurement of quantity *y* for patient *I* at day *j* if measured. The parameter model is $\beta_{i}\sim \text{log}N\left(0,\sigma_{\beta }^{2}\right)$, $b_{j}\sim N\left(b_{j-1},\sigma_{b}^{2}\right)$, for *j* > 1, $b_{j}\sim N\left(0,100^{2}\right)$ for *j* = 1 and $\sigma^{-2},\;\sigma_{\beta }^{-2},\;\sigma_{b}^{2}\sim \Gamma \left(0.01,\;0.01\right)$. Parameter estimates were calculated using Markov Chain Monte Carlo with 100,000 iterations and a thinning rate of 10 steps. The grand means for each time point were presented together with Bayesian 95% credible intervals. All statistical analyses were performed in the R statistical environment and Just Another Gibbs Sampler [22] linked by the ‘rjags’ package [23].

## Results

### Overall cohort

A total of 6,070 patients were admitted to our center during the study period for dengue. Selected demographic characteristics are presented in Table 1. The mean age of the cohort was 35.3 years (STD = 13.0 years). Male patients comprised 3927 (64.7%). Two hundred and ninety-six (4.9%) were elderly and 487 (8.0%) had hypertension. A total of 1,056 (17.4%) had SD. There were 1,599 (26.3%) who met the criteria for DHF. Warning signs were reported in 1,056 (17.4%). The patients presented to hospital at a median illness day of 5 days (interquartile range [IQR], 4–6). They were hospitalized for a median of 4 days (IQR, 3–5). Intensive care unit admission was required for 19 (0.3%). Intravenous fluid was given to 5,605 (92.3%). Platelet transfusion was given to 827 (13.6%) while blood transfusion was given to 35 (0.6%). Death occurred in 5 (0.1%).

**Table 1:** Patient characteristics.

		Dengue classifications			
	Total (n = 6,070)	Non-severe Dengue (n = 5,014)	Severe Dengue (n = 1,056)	Dengue Fever (n = 4,471)	Dengue Hemorrhagic Fever (n = 1,599)
**Mean age (Standard deviation)**	35.3 (13.0)	35.1 (12.9)	36.1 (13.5)	34.5 (12.9)	37.6 (13.0)
< 60 years	5,774 (95.1%)	4,787 (82.9%)	987 (17.1%)	4,276 (74.1%)	1,498 (25.9%)
≥ 60 years	296 (4.9%)	227 (76.7%)	69 (23.3%)	195 (65.9%)	101 (34.1%)
**Gender**					
Male	3,927 (64.7%)	3,465 (88.2%)	462 (11.8%)	2,997 (76.3%)	930 (23.7%)
Female	2,143 (35.3%)	1,549 (72.3%)	594 (27.7%)	1,474 (68.8%)	669 (31.2%)
**Hypertension**					
No	5,583 (92.0%)	4,641 (83.1%)	942 (16.9%)	4,136 (74.1%)	1,447 (25.9%)
Yes	487 (8.0%)	373 (76.6%)	114 (23.4%)	335 (68.8%)	152 (31.2%)

### Temporal trend of systolic and diastolic blood pressures

#### Severe dengue

**Fig 1:**
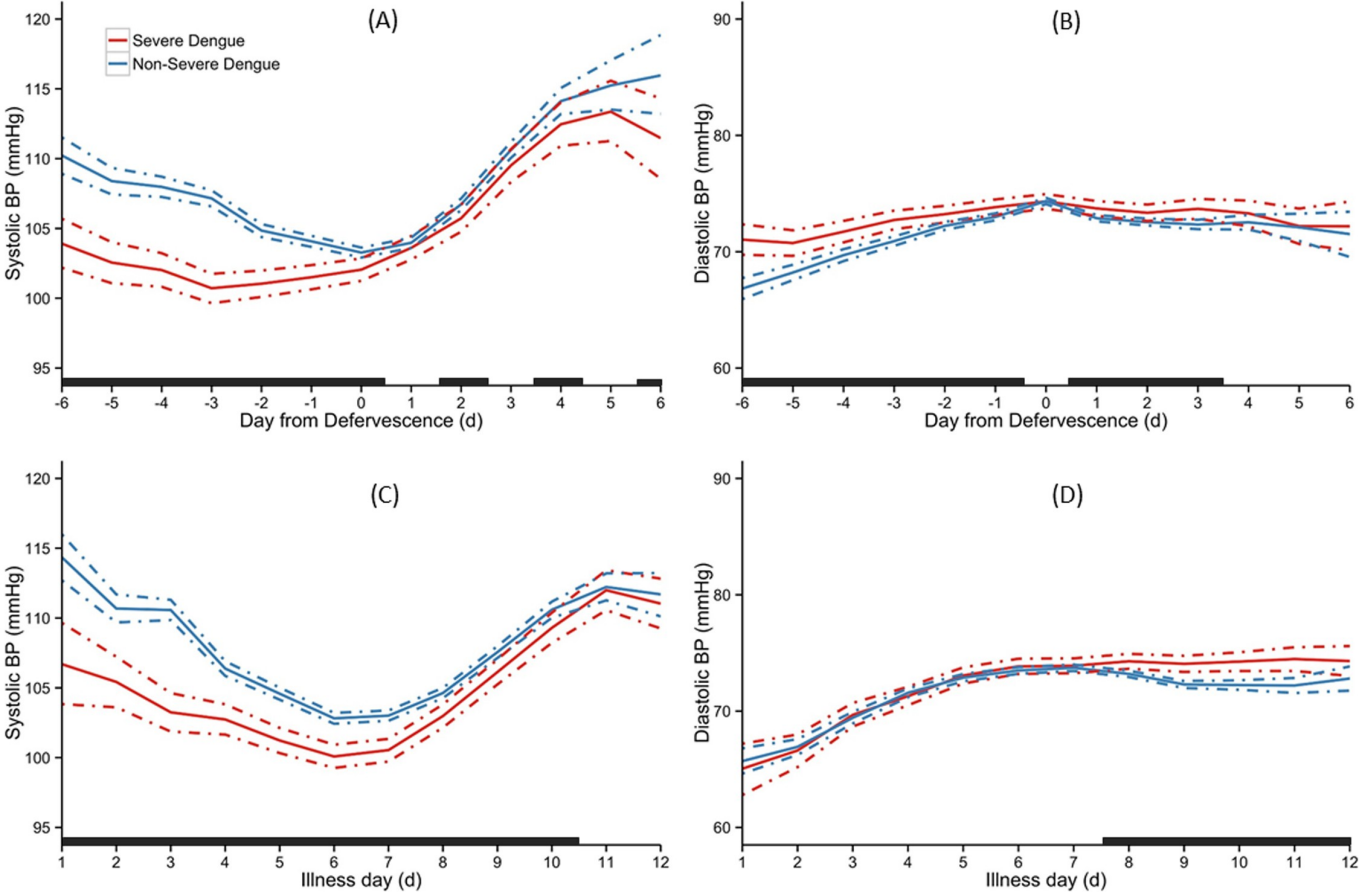
Temporal trend of SBP and DBP between patients with Severe Dengue and non-Severe Dengue. Overall means are indicated as solid lines with 95% credible intervals as dashed lines. The black bar on X-axis indicated days with a ‘‘significant” difference between the groups. The day of defervescence was denoted as day 0, day before defervescence as day -1, and day after defervescence as day 1 respectively, for SBP (A) and DBP (B). Day of onset of fever was denoted as illness day 0, for SBP (C) and DBP (D).

Fig 1 depicts the trend of SBP and DBP between patients with and without SD over the course of illness. Analyzed by day of defervescence, patients with SD had significantly lower SBP from day -6 to day +6, except days +1, +3 and +5 compared with those without (Fig 1A). Patients with SD had higher DBP from day -6 to day +6 except day 0 and days +4 to +6 compared with those without (Fig 1B). Analyzed by day of illness, patients with SD had lower SBP from day 1 to day 10 compared with those without (Fig 1C). DBP between the two groups did not differ significantly until days 8 to 12 of illness (Fig 1D).

#### Dengue hemorrhagic fever

**Fig 2:**
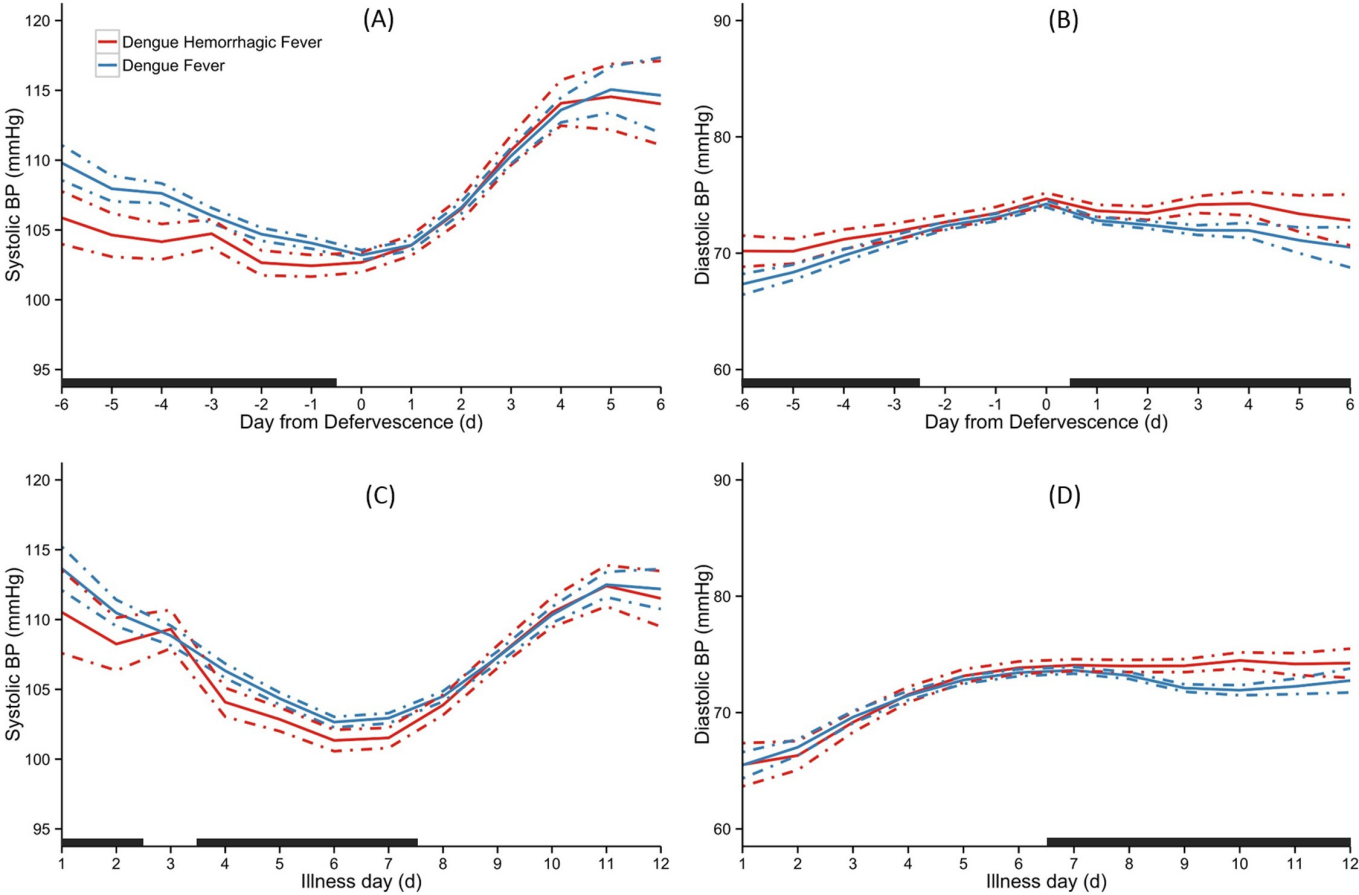
Temporal trend of SBP and DBP between patients with Dengue Hemorrhagic Fever and Dengue Fever. Overall means are indicated as solid lines with 95% credible intervals as dashed lines. The black bar on X-axis indicated days with a ‘‘significant” difference between the groups. The day of defervescence was denoted as day 0, day before defervescence as day -1, and day after defervescence as day 1 respectively, for SBP (A) and DBP (B). Day of onset of fever was denoted as illness day 0, for SBP (C) and DBP (D).

Fig 2 depicts the trend of SBP and DBP between patients with and without DHF over the course of illness. Analyzed by day of defervescence, patients with DHF had lower SBP from day -6 to day -1 but they had similar SBP compared with those without from the day of defervescence (Fig 2A). Patients with DHF had slightly higher DBP than patients without, through the course of illness except two days before and on the day of defervescence (Fig 2B). Patients with DHF had significantly lower SBP from day 1 to day 7 of illness, except day 3 compared with those without, with no difference in SBP thereafter (Fig 2C). Compared with those without, patients with DHF had similar DBP from day 1 to day 6, and higher DBP from day 7 to day 12 (Fig 2D).

**Fig 3:**
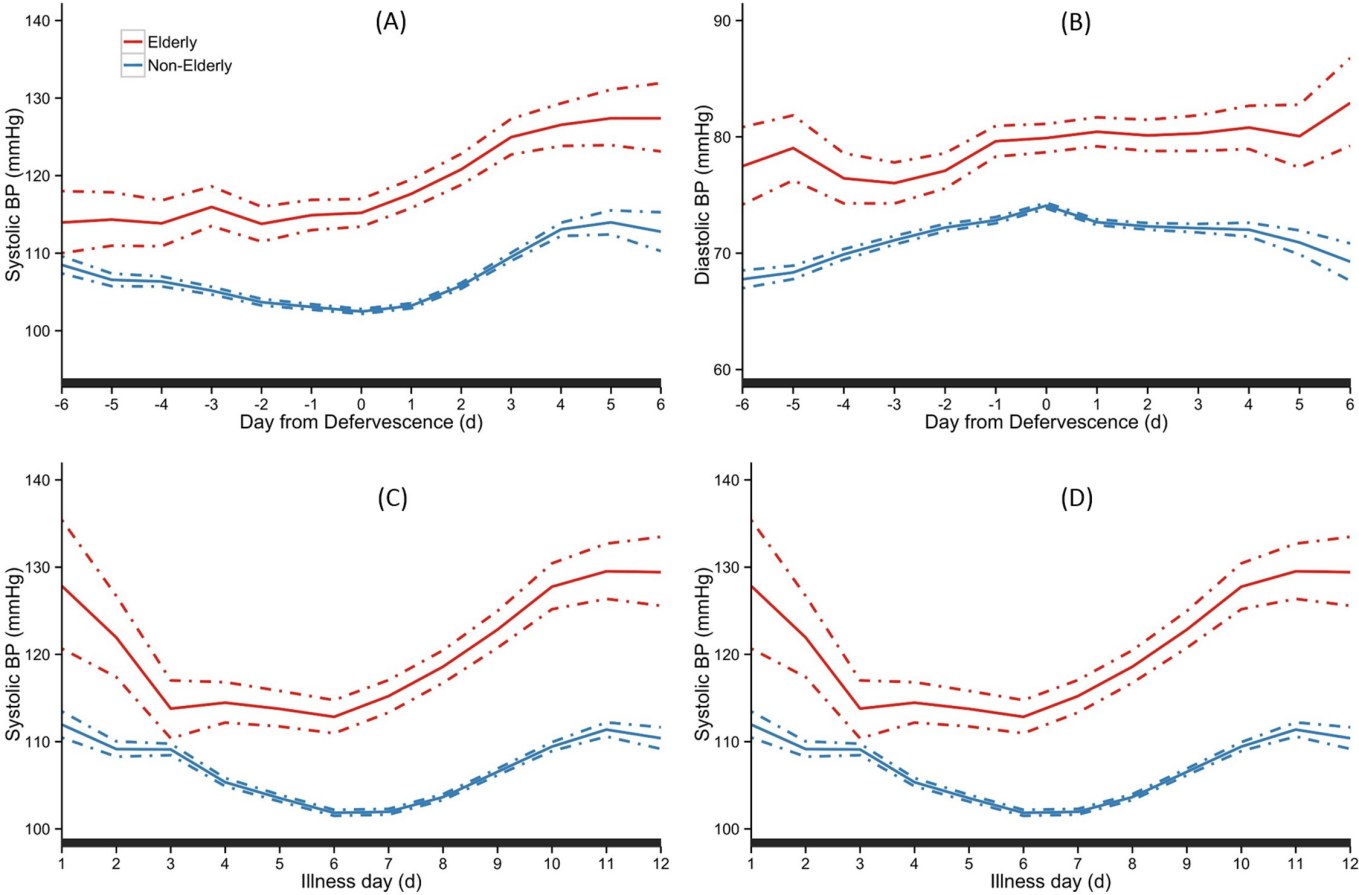
Temporal trend of SBP and DBP between the elderly and non-elderly. Overall means are indicated as solid lines with 95% credible intervals as dashed lines. The black bar on X-axis indicated days with a ‘‘significant” difference between the groups. The day of defervescence was denoted as day 0, day before defervescence as day -1, and day after defervescence as day 1 respectively, for SBP (A) and DBP (B). Day of onset of fever was denoted as illness day 0, for SBP (C) and DBP (D).

**Elderly patients.** Fig 3 depicts the trend of SBP and DBP between the elderly and non-elderly adults over the course of illness. When analyzed by day of defervescence, elderly patients maintained significantly higher SBP and DBP throughout the febrile, critical and recovery phases (Fig 3A and 3B). A similar pattern was observed when we examined the trend by day of illness (Fig 3C and 3D).

#### Hypertensive patients

**Fig 4:**
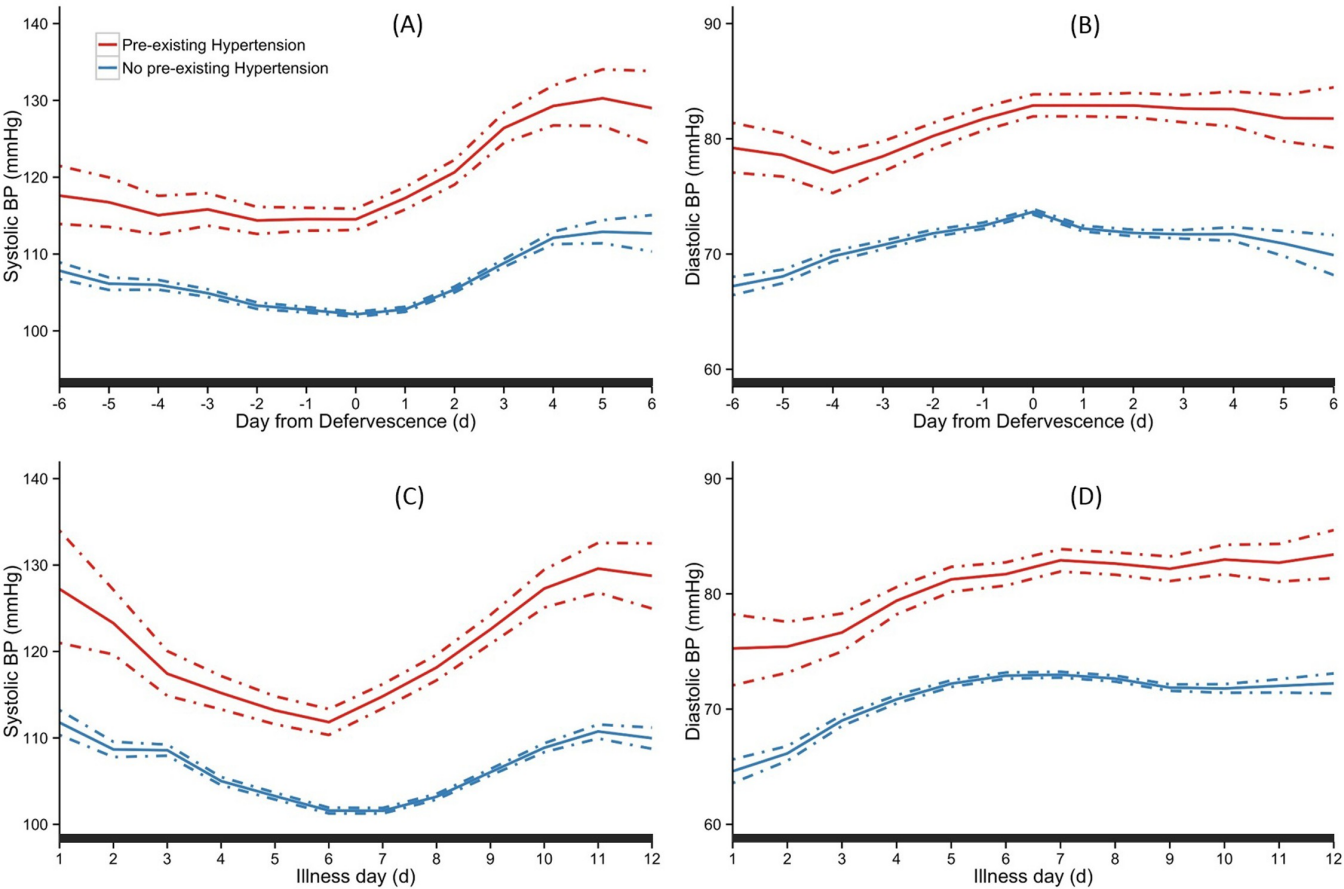
Temporal trend of SBP and DBP between patients with and without hypertension. Overall means are indicated as solid lines with 95% credible intervals as dashed lines. The black bar on X-axis indicated days with a ‘‘significant” difference between the groups. The day of defervescence was denoted as day 0, day before defervescence as day -1, and day after defervescence as day 1 respectively, for SBP (A) and DBP (B). Day of onset of fever was denoted as illness day 0, for SBP (C) and DBP (D).

Fig 4 depicts the trend of SBP and DBP between the patients with hypertension and those without over the course of illness. Patients with hypertension maintained higher SBP and DBP compared with patients without by day of defervescence. This trend was significant throughout the entire course of disease (Fig 4A and 4B). A similar pattern was observed by day of illness (Fig 4C and 4D).

## Discussion

There were small but significant differences in SBP trends between patients with SD or DHF, versus patients without SD or DHF, up till the day of defervesence. SBP decreased during disease progression, reaching a nadir around the 6th-7th day of illness corresponding to the day of defervesence and 1 day after. Recovery to a level exceeding that in the febrile phase was noted after days 2 or 3 post-defervescence. This corresponds to the accepted view [6] that plasma leakage occurs maximally in the ‘critical phase’ of dengue illness. There was no clinically meaningful difference between the two groups in terms of DBP. Average increases from the initial to peak DBP were less than 10 mmHg in all subgroups, including SD or DHF. This could be the evidence of narrowing of pulse pressure as the diastolic pressure rises towards the systolic pressure [3].

Changes to components of the capillary endothelial cell glycocalyx (eCG) layer has been implicated in the pathogenesis of plasma leakage [24, 25]. Hypertension is associated with increased sodium intake combined with genetic, hormonal and other factors [26–28]. Excess sodium has been linked to damage to the eCG *in vitro* [29, 30]. Direct damage to the eCG due to hypertension was observed in rats [31]. We postulate that these same eCG changes might also occur in the hypertensive subgroup of our sample. Moreover, there could also be involvement of renin-angiotensin system. Expression of renin in mouse and human cells after persistent cytomegalovirus infection caused an increase of arterial blood pressure [32]. Angiotensinogen was reported as predictive marker of shock in dengue. Following infection with dengue virus, there is up regulation of angiotensinogen and the high plasma levels of angiotensin II impair endothelial cell function and induce apoptosis of endothelial cells [33]. While the precise mechanism of increased capillary permeability and plasma leakage remains unknown, our findings suggest that possible chronic changes to the eCG from pre-existing hypertension does not act in the same direction as the acute changes seen in the dengue critical phase. There is uncertainty regarding the exact effect of eCG damage caused by hypertension as eCG damage in dengue tends to affect charge permeability with relative preservation of size permeability [34]. Further investigations at a molecular level are needed to elucidate the precise mechanisms causing this phenomenon.

Older adults showed smaller decrease in SBP compared with younger patients. As the prevalence of pre-existing hypertension among this age group has been estimated to be 75% in a large study in Singapore [15], the observation could be confounded by high rates of pre-existing hypertension which led to higher blood pressure trends throughout the duration of illness. Another possible explanation is that the elderly might mount a less aggressive immune response during dengue infection, leading to lower capillary permeability. Epidemiological observation in Cuba suggested that elderly patients with secondary dengue were not at increased risk of DHF [35]. Capillary leak in dengue infection results partly from immune activation; immunosenescence [36] may be protective in this setting.

Earlier report from Singapore [13] and Brazil [37] stated self-reported hypertension as risk factor for progression to DHF. Our study reported daily trends of recorded blood pressures. Our findings suggest that patients with pre-existing hypertension and elderly patients were significantly more likely to develop SD or DHF. However, both groups maintained higher SBP and DBP throughout the duration of illness. Clinicians caring for patients in these two subgroups should not be falsely reassured by relatively high blood pressure readings and must pay attention to other markers of severe disease such as signs of third spacing, bleeding or organ involvement.

Our study included a large sample size of adult dengue patients with frequent monitoring of blood pressure during hospitalization. This allowed for precise estimates of blood pressure trend. The use of a hierarchical model allowed for estimation of group-level effects whilst taking into account individual level effects. This study has a number of limitations. Blood pressure is influenced by the level of fluid replacement administered to patients. Provision of fluid therapy may mask the effects of plasma leakage and result in the small differences seen between patients with and without SD or DHF respectively. In addition, there may be differences in blood pressure parameters among dengue patients by serotypes. Therefore, more research is needed to study the impact of fluid therapy as well as infecting serotypes on blood pressure changes and dengue severity. Pre-existing hypertension was self-reported. In addition, we could not differentiate between patients who were well controlled on anti-hypertensive medications and patients who were poorly controlled due to the lack of data. However, anti-hypertensive medications were usually discontinued on admission.

In conclusion, we documented that in SD or DHF, SBP decreased to a nadir around the day of defervescence, and recovered to a level exceeding that in febrile phase by days 2 or 3 post-defervescence. Elderly patients and patients with pre-existing hypertension maintained higher SBP and DBP throughout the duration of dengue infection.
